# The Compartment Syndrome Associated with Deep Vein Thrombosis due to Rattlesnake Bite: A Case Report

**DOI:** 10.4274/balkanmedj.2016.0218

**Published:** 2017-08-04

**Authors:** Radu Ciprian Tincu, Zoie Ghiorghiu, Dana Tomescu, Radu Alexandru Macovei

**Affiliations:** 1 Toxicology-Intensive Care Unit, Bucharest Clinical Emergency Hospital, Bucharest, Romania; 2 Carol Davila University of Medicine and Pharmacy, Bucharest, Romania; 3 Anesthesiology Intensive Care Unit, Fundeni Clinical Institute, Bucharest, Romania

**Keywords:** Snakebite, Deep vein thrombosis, compartment syndrome

## Abstract

**Background:**

Snakebite is a health issue specific to some parts of the world, especially in the tropical areas, where it produces many victims. The main clinical damage caused by snakebite involves haemotoxic, neurotoxic and myotoxic reactions. We report the case of a young woman suffering from snakebite who developed deep vein thrombosis and compartment syndrome.

**Case Report:**

We present the case of a 32-year-old Romanian woman who was injured by her own Crotalinae snake (also known as pit viper or rattlesnake) on her left forearm. When admitted to our Emergency Department, she was conscious with a Glasgow coma scale of 12/15, somnolent, febrile, suffering of headache, tachypnoea; the marks of the snakebite were located in the distal part of the anterior left forearm; she had pain and bleeding at the bite site and swelling of the left upper limb with lymphangitis up to the axilla. She experienced fasciotomy-requiring compartment syndrome of the upper limb and required unfractionated heparin and close monitoring using activated partial thromboplastin time evolution due to micro-thrombosis in the brachial vein. Local improvement was achieved in the next 4 days with progressive diminishment of local tenderness and swelling.

**Conclusion:**

Limb deep vein thrombosis might be induced by snakebite, despite the pro-haemorrhagic general condition induced by the envenomation. A high index of clinical suspicion is needed for early diagnosis and timely management, which can improve survival of these patients.

Snakebite is a health issue specific to some parts of the world, especially in tropical areas, where it produces many victims. According to local epidemiology, in India there is a 30-fold higher level of deaths caused by snake bite than reported by scientific and medical reports (1). In Romania, only a small part of the territory includes snakes or vipers in its fauna. Bites usually occur accidently, on the foot or ankle as a result of stepping on the snake, or the hand if the person picks up the snake. The main clinical damage caused by snakebites involves a toxic reaction that is known to be haemotoxic, neurotoxic and myotoxic. It has also been established that the importance and magnitude of neurological impairment varies according to individual factors and is related to organ dysfunction, shock or hypotension. Although most of the local reactions are considered harmless, the haematological implications might be fatal due to pro-haemorrhagic reactions. Systemic haemorrhage and coagulopathy are rare phenomena in humans, because there is a small dose of venom distributed to a large body mass of the victim. Haematological abnormalities involve neutrophil leucocytosis, thrombocytopenia, anaemia, elevation of serum creatine phosphokinase, and metabolic acidosis (2). In the meantime, compartment syndrome after snakebite is considered to be quite rare, but has been reported involving the hand or forearm following envenomation (3). The emergency management may require surgical treatment, in order to save the nerves and soft tissues. Without intervention, venous and lymphatic drainage of the injured area will be compromised and secondary, the local arterioles will show induced tissue ischaemia and further lesions.

Classiclly, there are considered 5 envenomation grades, ranging from 0 to 4. Downey, Omer and Moneim described the severity of local reactions depending on the degree of envenomation as follows: grade 0 involves only swelling and erythema around the bite marks of less than 2.5 cm (no envenomation), grade 1 is considered when swelling and erythema are between 2.5 and 15 cm without clinical signs, grade 2 indicates swelling and erythema of 15 to 40 cm with only mild systemic signs, in grade 3 there is swelling and erythema over 40 cm with systemic signs, and grade 4 includes coma, shock, and severe systemic signs (4).

We report the case of a young woman suffering from snakebite who developed deep vein thrombosis and compartment syndrome. Informed consent was obtained from the patient in order to use medical record data.

## CASE PRESENTATION

**Figure 1:**
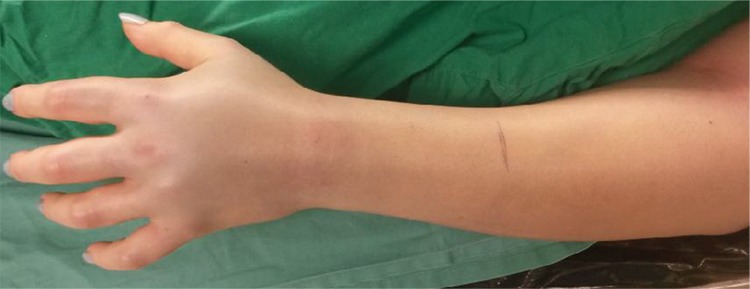
Increasing swelling of the left upper limb on the admission day.

We present the case of a 32-year-old Romanian woman, with no remarkable medical history, who was injured by her own Crotalinae snake (also known as a pit viper or rattlesnake) on her left forearm. She self-presented to the hospital within the first 45 minutes after the event; on her way to medical services, the forearm and hand developed important oedema, pain and paraesthesia. When she was presented to our Emergency Department, upon clinical examination, she was conscious with a Glasgow coma scale of 12/15, somnolent, febrile, and suffering from headache, and tachypnoea. The marks of the snakebite were located in the distal part of the anterior left forearm; she had pain and bleeding at the bite site and swelling of the left upper limb with lymphangitis up to the axilla, with local dolour and calour together with tender axillar and epitrohlear lymph nodes (Figure 1). Local pain was very severe and movements of the hand including fingers were also very painful; a diagnosis of compartment syndrome was made.

**Figure 2:**
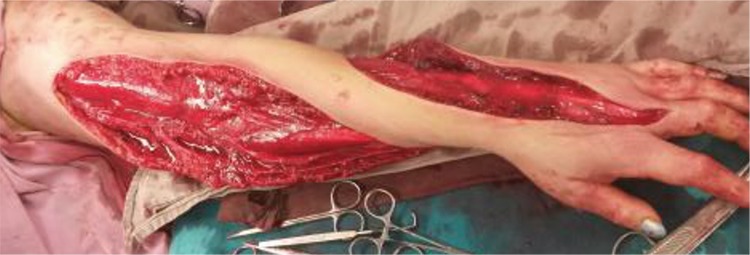
Extensive fasciotomy for compartment syndrome.

The urgent Doppler ultrasound evaluation of the left upper limb indicated non-compressibility of the brachial vein and micro-thrombosis on its lumen that enabled the diagnosis of deep vein thrombosis. The local neurological function was within normal range expected for paraesthesia of the median nerve. She was not administered polyvalent anti-snake venom because there was a lack of serum at that time in our centre and the surroundings. Concerning her laboratory findings, the haemoglobin was 15.3 mg/dL, white blood cell count was 16.700/mmc, with a left predominance, her coagulation profile revealed prolonged activated partial thromboplastin time (aPTT) and prothrombin time, and elevated D-Dimer and fibrinogen degranulation products plasma levels, but her renal and liver functions were normal. She was put on antibiotics treatment with piperacillin/tazobactum, linezolid and metronidazole. The local treatment required fasciotomy, and the evolution started to improve soon after the intervention (Figure 2).

**Figure 3:**
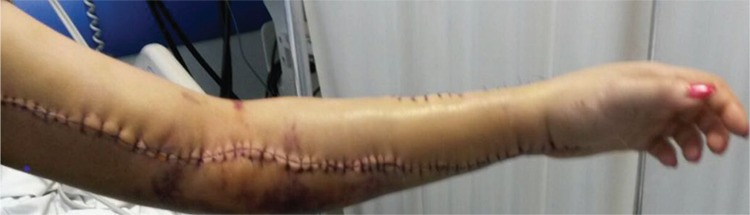
Improvement of local circulation and lesions in the 5th postoperative day.

The second day of post-operatory evolution revealed a reduction of the clinical signs of compartment syndrome. Considering the early onset of deep vein thrombosis, she received unfractionated heparin and was monitored using aPTT evolution. Local improvement was achieved in the next 4 days with progressive diminishment of local tenderness and swelling; soon, the movement was re-established (Figure 3).

Before discharge, the patient underwent repeated Doppler examination and the local flowmetry was normal with no micro thrombosis. She was prescribed warfarin under the regular evaluation of the international normalised ratio (INR).

## DISCUSSION

Snakebite is an uncommon pathology in Romania, although it is seen in some parts of the country or in professional or illegal snake growers. Nevertheless, it is mainly seen in tropical areas as a major cause of morbidity and mortality. The severity of clinical manifestations depends upon the degree of envenomation. The local aspect varies from swelling and different forms of cellulitis to systemic reactions with important cerebral or gastrointestinal bleeding. One of the most important complications involves haematological abnormalities, especially in viper bites. The coagulation cascade is activated because of two metalloproteinases contained in the venom (ecarin and carinactivase) that promote prothrombin activity (5).

There is a higher incidence of haemorrhagic events than thrombotic ones, and usually includes thrombocytopenia and prolonged bleeding. On the other hand, several thrombotic events have been reported: in a study from Martinique, authors encountered pulmonary embolism, cerebral infraction and myocardial infraction (6). A wide variation of proteolytic activities was found in the metalloproteinase composition of rattlesnake venom, counting haemorrhagic or thrombotic abnormalities (7,8).

In a study aimed to elucidate the molecular basis for intra-species variation of metalloproteinase-associated activities, Dagda et al. (9) modelled the three dimensional structures of four metalloproteinases based on the variations of the proteinase domain. The authors concluded that amino acid sequence variability within the N-terminal region of the mature proteinase domain, spatial deviations of the northern cleft wall, differences in the flexibility and size of the catalytic cleft along with molecular surface complexity and differences in the physicochemical environment surrounding the catalytic groove are the underlying factors for differences in the substrate selectivity of metalloproteinases (9).

Our case presented with deep vein thrombosis on the upper left limb and required systemic anticoagulant therapy with heparin, although some data from previous studies were discouraging (6). A similar case presenting left lower limb following snakebite, despite an ongoing coagulopathy, was described by Natarajan et al. (10). Although we could have expected multiple complications, the evolution was beneficial for the patient and we were able to discharge her on warfarin treatment, as the protocol indicates, for up to six months with regular monitoring of the INR. It was wise to promptly evaluate the patient with a Doppler ultrasound as the limb was losing its function, because this is the standard diagnostic tool for deep vein thrombosis. One might consider the rapid investigation of every patient with snakebite affecting one of the limbs using Doppler ultrasound, in order to prevent complications such as extended thrombosis.

Compartment syndrome occurring after snakebite has been previously reported (3). It imposes rapid decompression of all tissues in order to avoid future sequel, so doctors should pay attention to any of its clinical signs.

Limb deep vein thrombosis might be induced by snakebite, despite the pro-haemorrhagic general condition induced by envenomation. A high index of clinical suspicion is needed for early diagnosis and timely management, which can improve survival of these patients. Although many snakebite cases are asymptomatic and can be easily discharged, surgery should be applied to any other deep significant injuries along with close monitoring of local and systemic signs.

## References

[1] Mohapatra B, Warrell DA, Suraweera W, Bhatia P, Dhingra N, Jotkar RM, et al (2011). Snakebite mortality in India: a Nationally representative mortality survey. Plos Negl Trop Dis.

[2] Spiller HA, Bosse GM (2003). Prospective study of morbidity associated with snakebite envenomation. J Toxicol Clin Toxicol.

[3] Tucker SC, Josty I (2005). Compartment syndrome in the hand following an adder bite. J Hand Surg Br.

[4] Downey DJ, Omer GE, Moneim MS (1991). New Mexico rattlesnake bites: demographic review and guidelines for treatment. J Trauma.

[5] Cortelazzo A, Guerranti R, Bini L, Hope-Onyekwere N, Muzzi C, Leoncini R, et al (2010). Effects of snake venom proteases on human fibrinogen chains. Blood Transfus.

[6] Thomas L, Tyburn B, Bucher B, Pecout F, Ketterle J, Rieux D, et al (1995). Prevention of thromboses in human patients with Bothrops lanceolatus envenoming in Martinique: failure of anticoagulants and efficacy of a monospecific antivenom. Research Group on Snake Bites in Martinique.. Am J Trop Med Hyg.

[7] Dagda RK, Gasanov S, Rael ED, Lieb C (2013). Genetic basis for variation of metalloproteinase-associated biochemical activity in venom of the Mojave rattlesnake (Crotalus scutulatus). Biochem Rest Int.

[8] Massey DJ, Calvete JJ, Sanchez EE, Sanz L, Richards K (2012). Venom variability and envenoming severity outcomes of the Crotalus scutulatus (Mojave rattlesnake) from southern Arizona. J Proteomics.

[9] Dagda RK, Gasanov SE, Zhang B, Welch W, Rael ED (2014). Molecular models of the Mojave rattlesnake (Crotalus scutulatus scutulatus) venom metalloproteinases reveal a structural basis for differences in hemorrhagic activities. J Biol Phys.

[10] Natarajan N, Basheer A, Mookkappan S, Periyasamy S (2014). Reversible lower limb deep vein thrombosis following haemotoxic snakebite-a case report. AMJ.

